# Wnt Inhibition Safeguards Porcine Embryonic Stem Cells From the Acquisition of Extraembryonic Endoderm Cell Fates

**DOI:** 10.1002/advs.202416802

**Published:** 2025-03-10

**Authors:** Hanning Wang, Liang Zhong, Zhuangfei Wang, Jinzhu Xiang, Duanqing Pei

**Affiliations:** ^1^ Laboratory of Cell Fate Control School of Life Sciences Westlake University Hangzhou 310030 China; ^2^ Hebei Provincial Key Laboratory of Basic Medicine for Diabetes The Shijiazhuang Second Hospital Shijiazhuang 050051 China; ^3^ Westlake Laboratory of Life Sciences and Biomedicine Hangzhou 310030 China

**Keywords:** ESCs, pig, pluripotency, Wnt inhibition, XEN cells

## Abstract

Porcine embryonic stem cells (ESCs) are excellent models for exploring embryogenesis, producing genetically enhanced farm animals, and improving breeding. Various chemicals have been applied to generate porcine ESCs from embryos, which differ from mouse and human ESC derivation. Wnt inhibitors XAV939 or IWR1 are required to isolate and maintain porcine ESCs. How Wnt inhibitors specify porcine ESC fate decisions remains poorly understood. Additionally, whether porcine ESCs can be converted to extraembryonic endoderm (XEN) cells without genetic interventions has not been reported. Here, it is reported that Wnt inhibitors (i.e., XAV939 and IWR1) safeguard porcine ESCs from acquiring the XEN lineage. Porcine ESCs rely on Wnt inhibitors to maintain pluripotency. Without them, porcine ESCs exit from pluripotency and convert to XEN cells. An efficient strategy and culture conditions are further developed to directly derive porcine XEN cells from ESCs without gene editing. The resulting XEN cells from ESCs exhibit similar transcriptome and chromatin accessibility features to XEN cells from embryos and contribute to mouse extraembryonic tissues. This study will deepen the understanding of porcine pluripotency, lay the foundation for deriving high‐quality porcine ESCs with germline chimerism and transmission, and provide valuable materials to study extraembryonic development and lineage segregation in livestock.

## Graphical abstract

The study reports that Wnt inhibitors safeguard porcine embryonic stem cells (ESCs) from acquiring the extraembryonic endoderm (XEN) lineage, and further develop an efficient strategy to derive XEN cells from ESCs. The XEN cells from ESCs exhibit similar transcriptome and chromatin accessibility features to XEN cells from embryos and contribute to mouse extraembryonic tissues. This study will deepen the understanding of porcine pluripotency and provide valuable materials to study extraembryonic development in livestock.

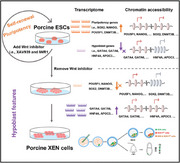

## 1 Introduction

The pig is considered a preferable model for studying human disease and a very suitable surrogate for humanized organs as it shares genetic, physiological, and anatomical similarities with humans.^[^ ^1^ ^]^ During porcine early embryonic development, inner cell mass (ICM) and the trophectoderm specification occur at 5 days post‐fertilization (dpf); the epiblast and hypoblast (HYPO or primitive endoderm/PrE) are then specified from ICM at 6 dpf.^[^ ^2^ ^]^ Stem cells have been in vitro derived from ICM, epiblasts, and HYPO, which serve as excellent models for biomedical research and animal breeding for agriculture and animal husbandry.

Porcine pluripotent stem cells (PSCs) have been generated from ICMs,^[^ ^3^, ^4^ ^]^ epiblasts,^[^ ^5^, ^6^ ^]^ or whole blastocysts^[^ ^7^, ^8^, ^9^ ^]^ under different culture conditions. Extraembryonic endoderm stem (XEN) cells can be isolated from blastocysts.^[^ ^10^, ^11^, ^12^ ^]^ Despite applying various chemical inhibitors and different combinations to generate porcine embryonic stem cells (ESCs),^[^ ^7^, ^8^, ^9^ ^]^ EPSCs (expanded potential stem cells),^[^ ^3^, ^4^ ^]^ EDSCs (embryonic disc stem cells),^[^ ^5^ ^]^ and pgEpiSCs (pig epiblast stem cells),^[^ ^6^ ^]^ Wnt inhibitors XAV939^[^ ^3^, ^4^, ^5^, ^7^ ^]^ or IWR1^[^ ^6^, ^7^, ^8^, ^9^ ^]^ are required to isolate and maintain porcine PSCs, which are different from human and mouse PSC generation. For instance, the removal of IWR1 reduced cell proliferation and promoted differentiation of porcine ESCs,^[^ ^8^ ^]^ as well as, down‐regulated pluripotency marker expression and up‐regulated mesoderm and endoderm‐related marker expression of pgEpiSCs.^[^ ^6^ ^]^ The withdrawal of XAV939 led to the collapse of outgrowth proliferation and OCT4 negative expression in porcine EPSCs,^[^ ^3^, ^4^ ^]^ and down‐regulated pluripotency markers OCT4 and SOX2 expression in porcine EDSCs.^[^ ^5^ ^]^ In mice, the combination of XAV939 and CHIR99021 supports mouse epiblast stem cells (EpiSCs) in undifferentiated states.^[^ ^13^ ^]^ Administration of XAV939 or IWR1 promotes the derivation of mouse EpiSCs.^[^ ^14^, ^15^ ^]^ In human naïve PSCs, the presence of XAV939 contributes to a more primed‐like protein expression profile^[^ ^16^ ^]^ or more responsive for differentiation signals.^[^ ^17^ ^]^ While, for human naïve PSCs cultured in the PXGL medium,^[^ ^18^ ^]^ XAV939 reduced trophectoderm markers GATA2 and GATA3 expression.^[^ ^19^ ^]^

In our recent study, we developed the 4FIXY medium to isolate and culture porcine ESCs.^[^ ^7^ ^]^ Chemicals XAV939 and IWR1 are essential for deriving porcine ESCs from blastocysts.^[^ ^7^ ^]^ If XAV939 and IWR1 are removed from the porcine ESC culture conditions, i.e., 4FIXY, how porcine ESC fate changes has not been properly characterized. Elucidating cell fate decisions in this process sets a framework for defining the signaling network of porcine pluripotency and contributes to optimizing porcine ESC culture condition. Here, we show that the withdrawal of XAV939 and IWR1 causes porcine ESCs to exit from pluripotency and potentially drives them toward XEN cells. We establish a novel method and an efficient culture condition (termed 2FYSC) that supports the derivation of porcine XEN cells from ESCs and blastocysts. The porcine XEN cells maintain stable morphology, transcriptome, karyotype, and developmental potentials. Our findings not only allow us to understand the porcine ESC pluripotency maintenance and exit, but also provide a platform to study porcine extraembryonic development and function.

## 2 Results

### 2.1 Removal of XAV939 and IWR1 Leads to the Exit From Pluripotency in Porcine ESCs

**Figure 1:**
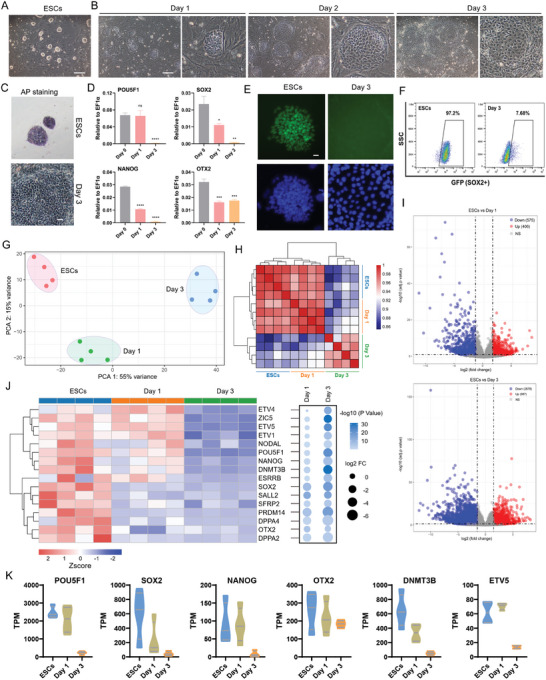
The exit from pluripotency in porcine ESCs regulated by XAV939 and IWR1. A) Representative morphology of porcine ESCs cultured in the 4FIXY medium. Scale bar, 200 µm. B) Representative morphology of cells cultured in the 4FY medium. Scale bar, 200 µm. C) AP staining. Scale bar, 50 µm. D) Real‐time quantitative PCR data of pluripotency markers following 0 (ESCs), 1, and 3 days of exposure to the 4FY culture condition. *n* = 3. The data are presented as the mean ± SD. Day 1 vs. Day 0; Day 3 vs. Day 0. ^*^ *p*<0.05; ^**^ *p*<0.01; ^***^ *p*<0.001; ^****^ *p*<0.0001. The *p* values were calculated using Student's *t*. E) Representative immunofluorescent staining for pluripotency markers SOX2. Scale bar, 20 µm. F) Flow cytometric analysis of SOX2 positive cells following 3 days of treatment in 4FY medium. G) Principal component analysis (PCA) of bulk RNA‐Seq data of ESCs in 4FIXY and cells in 4FY for 1 and 3 days. H) Correlation analysis among ESCs in 4FIXY and cells in 4FY for 1 and 3 days. I) Volcano plots of differentially expressed genes (DEGs) between ESCs in 4FIXY and cells in 4FY for 1 or 3 days. log2 fold change >1.5; adj. *p* < 0.05. J) Heatmap showing pluripotent genes among ESCs and cells in 4FY. Bubble plots indicating the fold change and statistical significance for the indicated genes (Day 1/3 vs. ESCs). K) RNA‐Seq expression (TPM values) of pluripotent genes.

We recently showed that porcine ESCs can be generated from blastocysts using a 4FIXY medium, including four cytokines and three chemical inhibitors Y27632, XAV939, and IWR1.^[^ ^7^ ^]^ In porcine PSC culture and maintenance, the chemical inhibitors XAV939^[^ ^3^, ^4^, ^5^, ^7^, ^8^ ^]^ and IWR1^[^ ^6^, ^7^ ^]^ are required to maintain the pluripotency of porcine ESCs, which is very different in mice and humans. When removing XAV939 and IWR1 from the 4FIXY medium (termed 4FY medium), dense ESC colonies with smooth and clear edges became flat and incompact, similar to XEN cells (**Figure** **1**A,B). The alkaline phosphatase (AP) staining became negative (Figure 1C). Real‐time quantitative PCR showed that the removal of XAV939 and IWR1 down‐regulated pluripotency genes^[^ ^2^, ^20^ ^]^ *POU5F1*, *SOX2*, *OTX2*, and *NANOG* (Figure 1D). By immunofluorescent staining, we did not detect the presence of pluripotency marker SOX2 (Figure 1E). Little SOX2‐GFP expression was recorded when cultured in 4FY medium for 3 days (Figure 1F). Additionally, we performed bulk RNA‐sequencing (RNA‐Seq) and comprehensively analyzed the global transcriptional profiles. The principal component analysis (PCA) revealed a distinct gene expression pattern between ESCs in the 4FIXY medium and cells in the 4FY medium for 1 and 3 days (Figure 1G). Pearson correlation analysis revealed a close relationship between ESCs and cells in the 4FY medium for 1 day compared with cells in the 4FY medium for 3 days (Figure 1H). The volcano plot showed 981 differentially expressed genes (DEGs) between ESCs and cells in the 4FY medium for 1 day and 3665 DEGs between ESCs and cells in the 4FY medium for 3 days (Figure 1I). Pluripotency genes^[^ ^2^, ^20^ ^]^ (i.e., *POU5F1*, *SOX2*, *ETV5*, and *NANOG*) were down‐regulated in cell populations in the 4FY medium for 1 and 3 days (Figure 1J,K). KEGG analysis showed that down‐regulated genes in 4FY were mainly related to signaling pathways regulating the pluripotency of stem cells (Figure S1A, Supporting Information). Taken together, porcine ESCs exit from pluripotency after withdrawing XAV939 and IWR1 in porcine ESC culture conditions.

### 2.2 Porcine ESCs Exhibit Increased XEN Potential After the Withdrawal of XAV939 and IWR1

**Figure 2:**
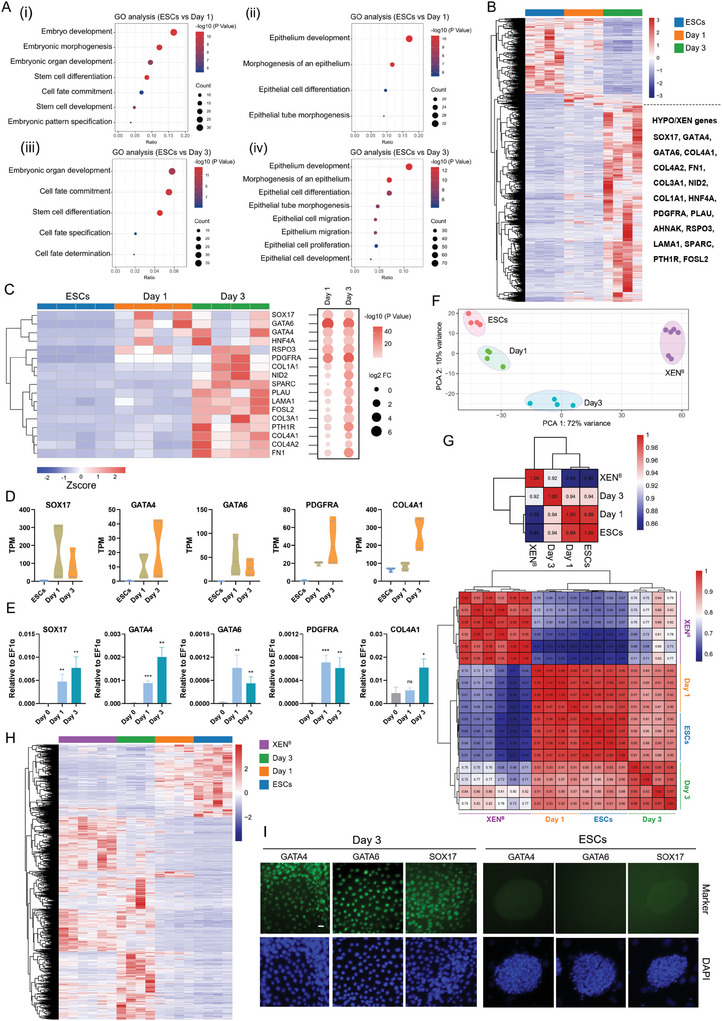
Porcine ESCs have the potential to transform into XEN cells. A) GO enrichment analysis of up‐regulated genes following treatment in 4FY. B) Heatmap showing DEGs between ESCs and cells in 4FY for 3 days. C) Heatmap indicating the expression of HYPO/XEN genes. Bubble plots showing the fold change and statistical significance for the indicated genes (Day 1/3 vs. ESCs). D) RNA‐Seq expression (TPM values) of HYPO/XEN genes of ESCs in 4FIXY and cells in 4FY for 1 and 3 days. E) Real‐time quantitative PCR showing the expression of HYPO/XEN genes (*SOX17*, *GATA4*, *GATA6*, *PDGFRA*, and *COL4A1*) of ESCs in 4FIXY and cells in 4FY for 1 and 3 days. *n* = 3. The data are presented as the mean ± SD. Day 1 vs. Day 0; Day 3 vs. Day 0. ^*^ *p*<0.05; ^**^ *p*<0.01; ^***^ *p*<0.001. The *p* values were calculated using Student's *t*. F) PCA of bulk RNA‐Seq data of ESCs in 4FIXY, cells in 4FY for 1 and 3 days, and XEN cells derived from blastocysts. G) Correlation analysis among ESCs in 4FIXY, cells in 4FY for 1 and 3 days, and XEN cells from blastocysts. H) Transcriptional profile based on DEGs between ESCs and cells in 4FY for 3 days. I) Representative immunofluorescent staining for markers of HYPO/XEN (SOX17, GATA4, and GATA6) in cells cultured in 4FY and ESCs maintained in 4FIXY. Scale bar, 20 µm. XEN^B^: XEN cells from blastocysts.

**Figure 3:**
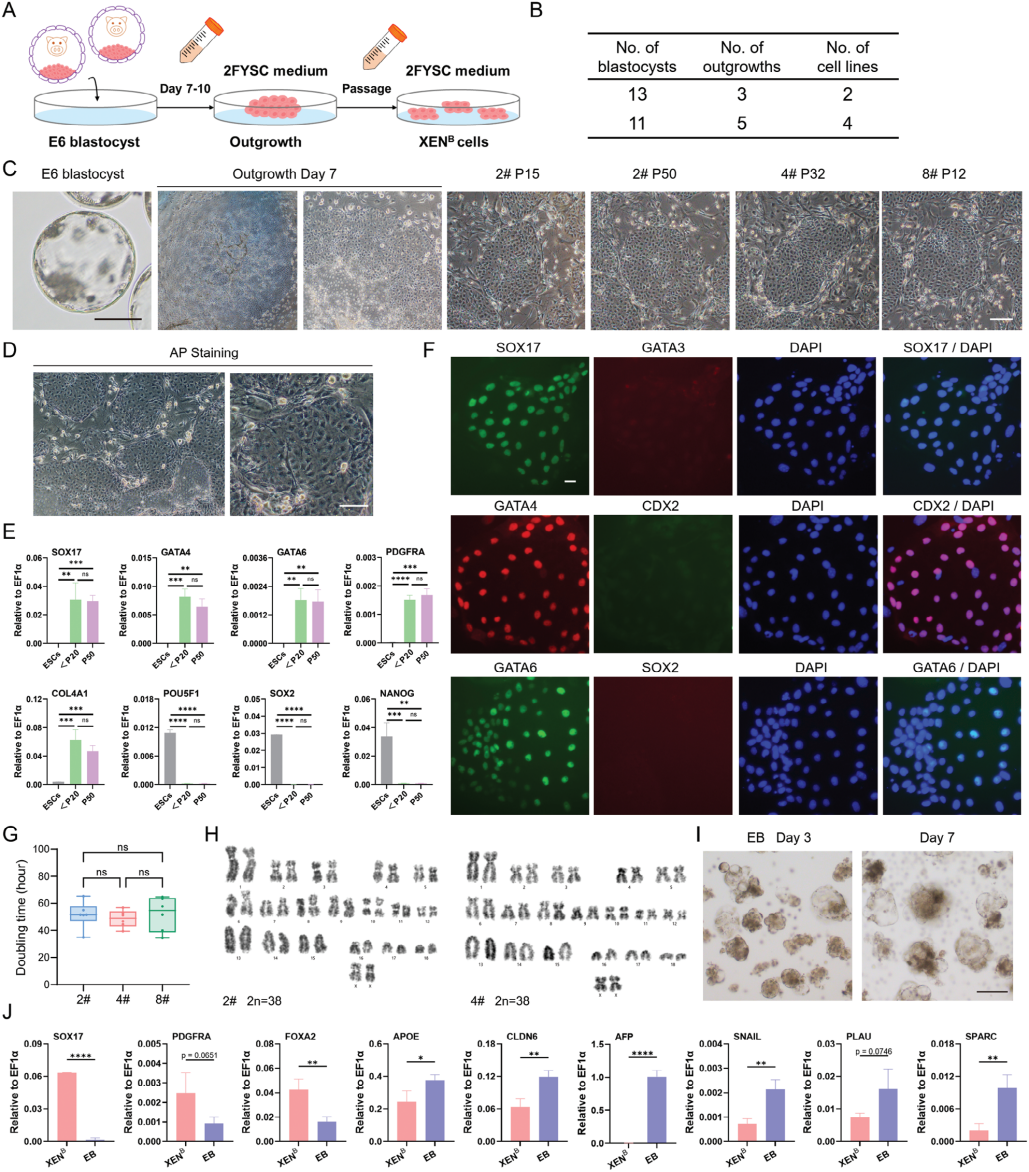
Derivation and characterization of porcine XEN cells from blastocysts. A) Schematic of the derivation of porcine XEN cells from blastocysts. B) Summary of derivation of XEN cell lines in 2FYSC culture medium. C) Representative morphology of outgrowth and XEN cells. D) AP staining of XEN cells. Scale bars, 100 µm. E) Real‐time quantitative PCR showing the expression of HYPO/XEN genes (*SOX17*, *GATA4*, *GATA6*, *PDGFRA*, and *COL4A1*) and pluripotency genes (*POU5F1*, *SOX2*, and *NANOG*). The <P20 and P50 indicate XEN cells with a passage less than 20 and passage 50, respectively. *n* = 3. The data are presented as the mean ± SD. <P20 vs. ESCs; P50 vs. ESCs. ns, no significance;^*^ *p*<0.05; ^**^ *p*<0.01; ^***^ *p*<0.001; ^****^ *p*<0.0001. The *p* values were calculated using Student's *t*. F) Representative immunofluorescent staining for markers of HYPO/XEN (SOX17, GATA4, and GATA6), trophoblast (CDX2 and GATA3), and epiblast (SOX2). Scale bar, 20 µm. G) Doubling time of different XEN cell lines. *n* = 6. The data are presented as the mean ± SD. One‐way ANOVA was used to assess statistically significant. ns, no significance. H) Karyotypes analysis of XEN cells. I) EB formation of XEN cells. Scale bar, 50 µm. J) Real‐time quantitative PCR showing the relative expression of several ParE and VE markers. XEN^B^: XEN cells from blastocysts. *n* = 3. The data are presented as the mean ± SD. ^*^ *p*<0.05; ^**^ *p*<0.01; ^***^ *p*<0.001; ^****^ *p*<0.0001. The p values were calculated using Student's *t*.

Next, we investigated how the cell fate changes after the exit from pluripotency regulated by XAV939 and IWR1. Up‐regulated genes in the 4FY medium for 1 and 3 days were enriched in gene ontology (GO) terms associated with embryo development, stem cell fate commitment, and cell fate specification, giving us a hint of cell fate changes of porcine ESCs (**Figure** **2**A). Down‐regulated genes were related to sodium ion homeostasis, regulation of membrane potential, and synaptic signaling. KEGG analysis showed that up‐regulated genes in 4FY were enriched in the PI3K‐Akt signaling pathway, Wnt signaling pathway, and Focal adhesion (Figure S1B–D, Supporting Information). It is widely known that XEN cells are generated from PrE or HYPO that is a mainly extraembryonic epithelium arising from the ICM of the mammalian pre‐implantation blastocyst.^[^ ^12^, ^21^ ^]^ Interestingly, GO analysis of up‐regulated genes in the 4FY medium for 1 and 3 days showed the enrichment of GO terms related to epithelium development, morphogenesis of an epithelium, epithelial cell development, and positive regulation of epithelial cell migration (Figure 2A), indicating the conversion of ESCs to XEN cells. Consistently, the heatmap showed the up‐regulation of HYPO‐related genes^[^ ^2^, ^20^ ^]^ including *SOX17, GATA4*, *GATA6*, *PDGFRA*, and *COL4A1* in cell populations cultured in the 4FY medium for 1 and 3 days (Figure 2B–D). Real‐time quantitative PCR corroborated these data (Figure 2E). We then compared these cells to porcine XEN cells from blastocysts (**Figure** **3**A) and performed a comparative analysis of the transcriptional profiles among these cells. In the correlation analysis, correlation coefficients between ESCs/cells in 4FY for 1 day and XEN cells ranged from 0.56 to 0.72, while correlation coefficients between cells in 4FY for 3 days and XEN cells were between 0.68 and 0.86 (Figure 2G, down). Similarly, the correlation coefficient between cells in 4FY for 3 days and XEN cells is 0.92, which is higher than that between ESCs/cells in 4FY for 1 day and XEN cells (0.85/0.86) (Figure 2G, up). These data indicate that porcine XEN cells show a close correlation with cells in 4FY for 3 days compared to ESCs and cells in 4FY for 1 day, and ESCs show a close relationship with cells cultured in 4FY for 1 day (Figure 2F,G). Based on DEGs between ESCs and cells in 4FY for 3 days, the heatmap was analyzed and showed the transcriptional similarities between cells in 4FY for 3 days and XEN cells from blastocysts (Figure 2H). By immunofluorescent staining, we detected the presence of GATA4, GATA6, and SOX17 in cells cultured in 4FY for 3 days (Figure 2I). These results indicate that porcine ESCs potentially transform into XEN cells after withdrawing XAV939 and IWR1 in porcine ESC culture conditions.

It has been reported that XAV939^[^ ^5^ ^]^ and IWR1^[^ ^6^ ^]^ are the Wnt inhibitors. Next, we tested whether the addition of the Wnt activator can result in the exit from pluripotency and potentially convert into XEN cells. Porcine ESCs were cultured in the 4FIXY medium plus CHIR99021 with a high concentration (3 µM). With the addition of CHIR99021, two typical morphologies were formed, one being dense colonies that are AP positive and the other being flat and in compact cells that are AP negative (Figure S2A,B, Supporting Information). Real‐time quantitative PCR showed that adding CHIR99021 caused a slight reduction of pluripotency genes *POU5F1*, *SOX2*, *OTX2*, and *NANOG*, and an increase of HYPO/XEN marker genes *SOX17, GATA4*, *GATA6*, and *PDGFRA* (Figure S2C, Supporting Information). We also cultured ESCs in the 4FY medium plus CHIR99021 with a high concentration. Similar to cells in the 4FY medium, compact colonies became flat and monolayer that are AP weakly positive or negative (Figure S2D,E, Supporting Information). Pluripotency genes were down‐regulated and HYPO/XEN markers were up‐regulated (Figure S2F, Supporting Information). We also compared the XEN and pluripotency genes between cells in 4FIXY+CHIR99021 and 4FY+CHIR99021 for 3 days. The data showed that, compared to cells in 4FIXY+CHIR99021, the expression of XEN genes (i.e., *SOX17*, *GATA4*, and *GATA6*) was higher, while the expression of pluripotency genes (i.e., *POU5F1*, *SOX2*, and *NANOG*) was lower in cells in 4FY+CHIR99021 (Figure S2G, Supporting Information), indicating that the withdraw of XAV939 and IWR1 enhances the XEN lineage potential. Taken together, these results indicate that the withdrawal of XAV939 and IWR1 or the addition of CHIR99021 leads to the pluripotency loss and the increased XEN lineage potential of porcine ESCs.

### 2.3 Derivation of Porcine XEN Cells From Blastocysts Using a Novel Culture Condition

To investigate whether XEN cells can be established from porcine ESCs cultured in the absence of XAV939 and IWR1, we first attempted to develop a robust system to derive XEN cells from porcine embryos, given that published porcine XEN culture conditions^[^ ^10^, ^11^, ^12^ ^]^ are suitable for cell passaging as clumps but not at single cells, which may cause inconvenience. To this end, we developed a novel culture condition (called 2FYSC), including cytokines EGF and bFGF and chemical inhibitors Y27632, SB431542, and CHIR99021 (Figure 3A,B). The resulting XEN cells can be passaged at single cells by enzymatic dissociation every 3‐4 days and form flat monolayer colonies (Figure 3C), which were AP negative (Figure 3D). During long‐term culture, these porcine XEN cells retain robust proliferative potentials (>50 passages) (Figure 3C). Real‐time quantitative PCR showed that HYPO marker genes (i.e., *SOX17*, *GATA4*, *GATA6*, *PDGFRA*, and *COL4A1*) were expressed in XEN cells, whereas pluripotency genes (i.e., *POU5F1*, *SOX2*, and *NANOG*) were minimally expressed (Figure 3E). Immunofluorescent staining showed that XEN cells expressed HYPO markers SOX17, GATA4, and GATA6, but not pluripotency marker SOX2 and TE markers CDX2 and GATA3 (Figure 3F). Different XEN cell lines exhibited similar doubling time and retained normal karyotypes (Figure 3G,H). Furthermore, XEN cells can form embryonic bodies (EBs) in suspension culture in vitro (Figure 3I). After random differentiation through EBs for 7 days, some HYPO or XEN‐related genes *SOX17*, *PDGFRA*, and *FOXA2* were down‐regulated, while several visceral endoderm (VE) and parietal endoderm (ParE)‐related genes *APOE*, *CLDN6*, *AFP*, *SNAIL*, *PLAU*, and *SPARC* were up‐regulated (Figure 3J). Overall, porcine XEN cells are generated from blastocysts using the 2FYSC medium.

### 2.4 Generation of XEN Cells From ESC‐Derived Intermediates Mediated by Removing Wnt Inhibitors

**Figure 4:**
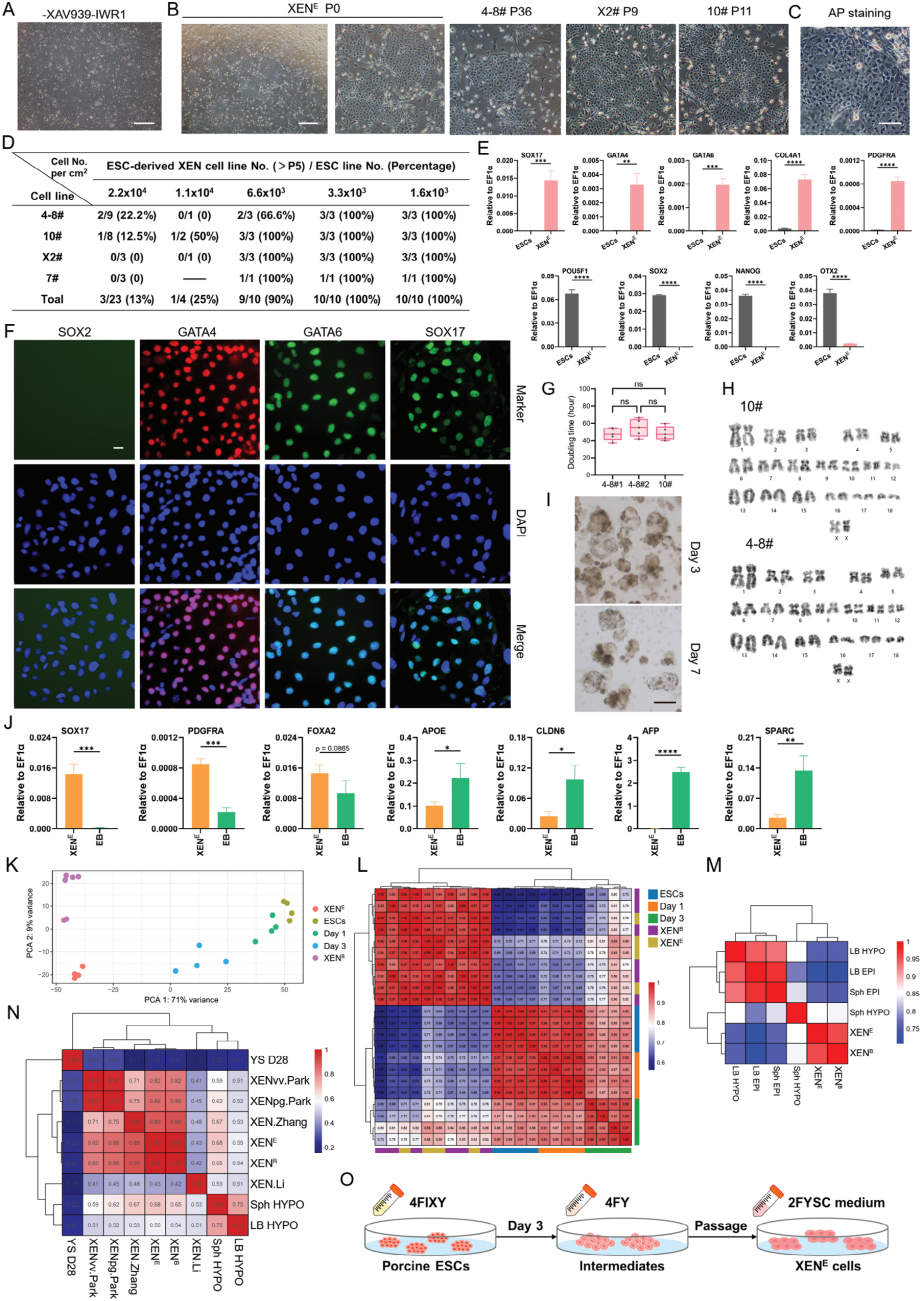
Conversion of porcine XEN cells from porcine ESC‐derived intermediates. A) Continuous culture in the 4FY medium after passaging of cells in 4FY for 3 days. Scale bar, 200 µm. B) Representative morphology of XEN cells generated from ESCs. Scale bar, 500 µm. C) AP staining of XEN cells from ESCs. D) Summary of the conversion of XEN cells from ESCs. E) Real‐time quantitative PCR showing the expression of HYPO/XEN genes (*SOX17*, *GATA4*, *GATA6*, *PDGFRA*, and *COL4A1*) and pluripotency genes (*POU5F1*, *SOX2*, *OTX2*, and *NANOG*). *n* = 3. The data are presented as the mean ± SD. ^**^ *p*<0.01; ^***^ *p*<0.001; ^****^ *p*<0.0001. The p values were calculated using Student's *t*. F) Representative immunofluorescent staining for markers of HYPO/XEN (SOX17, GATA4, and GATA6). Scale bar, 20 µm. G) Doubling time of different XEN cell lines. *n* = 5. The data are presented as the mean ± SD. One‐way ANOVA was used to assess statistically significant. ns, no significance. H) Karyotypes analysis of XEN cells. I) EB formation of XEN cells. Scale bar, 100 µm. J) Real‐time quantitative PCR showing the relative expression of several ParE and VE markers. *n* = 3. The data are presented as the mean ± SD. ^*^ *p*<0.05; ^**^ *p*<0.01; ^***^ *p*<0.001; ^****^ *p*<0.0001. The *p* values were calculated using Student's *t*. K) PCA of bulk RNA‐Seq data of ESCs in 4FIXY, cells in 4FY for 1 and 3 days, XEN cells derived from blastocysts and transformed from ESCs. L) Correlation analysis among ESCs in 4FIXY, cells in 4FY for 1 and 3 days, XEN cells derived from blastocysts and transformed from ESCs. M) Correlation analysis among XEN cells derived from blastocysts and transformed from ESCs, and published porcine embryos.^[^ ^20^ ^]^ N) Correlation analysis among XEN cells from blastocysts and ESCs, published XEN cells,^[^ ^10^, ^11^, ^12^ ^]^ porcine hypoblasts,^[^ ^20^ ^]^ and day 28 pig yolk sac (YS D28).^[^ ^12^ ^]^ O) Schematic of the derivation of porcine XEN cells from ESCs. XEN^E^: XEN cells from ESCs. XEN^B^: XEN cells from blastocysts. LB: late blastocyst; Sph: spherical embryo; EPI: epiblast; HYPO: hypoblast.

We tested whether ESC‐derived cells in the 4FY medium for 3 days can be successfully converted to XEN cells that are stably and consistently passaged in culture. First, we passaged ESC‐derived cells in the 4FY medium for 3 days and continued culturing them using the 4FY medium. However, cell colonies cannot normally proliferate (**Figure** **4**A), indicating that culture conditions are not suitable for cell growth and survival for a long time. Second, the 2FYSC medium for isolating XEN cells from blastocysts was used to culture ESC‐derived cells after passaging. In the 2FYSC medium, the flat and monolayer cell colonies, like XEN cells, were gradually formed (Figure 4B). These XEN‐like cells from ESCs can stably proliferate and passage as single cells, and the AP staining was negative (Figure 4C). About four ESC lines were used to derive XEN cells (Figure 4D). The proper cell density of ESCs seeded on the feeder cells is important for successfully generating XEN cells. Additionally, porcine ESCs were directly transferred and cultured from 4FIXY to 2FYSC medium for 3 days. The typical flat and monolayer cells cannot be well formed and most of these cells were SOX17 negative (Figure S3, Supporting Information), indicating that XEN cells were not formed using this method. Taken together, a two‐step conversion strategy is required for the establishment of porcine XEN cells from ESCs, the first step being cultured in the 4FY medium to form the intermediates with the increased XEN potential and the second step being cultured in the 2FYSC medium to generate stable porcine XEN cells.

Next, we detected the characteristics of these porcine XEN cells converted from ESCs. Compared with porcine ESCs, HYPO/XEN‐related genes *SOX17*, *GATA4*, *GATA6*, *PDGFRA*, and *COL4A1* were up‐regulated, while pluripotency genes *POU5F1*, *SOX2*, *OTX2*, and *NANOG* were down‐regulated in porcine XEN cells (Figure 4E). Immunofluorescent staining showed the presence of GATA4, GATA6, and SOX17 but not SOX2 (Figure 4F). During long‐term culture, cells have a similar doubling time with XEN cells from blastocysts and keep normal karyotypes (Figure 4G,H). When XEN cells were digested as single cells and cultured in ultra‐low attachment plates, the EB‐like structures gradually formed and were collected after random differentiation for 7 days (Figure 4I). Real‐time quantitative PCR showed an increase of VE and ParE‐related genes *APOE*, *CLDN6*, *AFP*, *SNAIL*, *PLAU*, and *SPARC*, and a decrease of HYPO or XEN genes *SOX17*, *PDGFRA*, and *FOXA2* (Figure 4J). To determine the transcriptional profiles of porcine XEN cells, we performed bulk RNA‐Seq of XEN cells from blastocysts and ESCs. PCA and correlation analysis showed that XEN cells transformed from ESCs bear similarity to XEN cells derived from blastocysts (Figure 4K,L). We then compared these cells to porcine early embryos^[^ ^20^ ^]^ and other published porcine XEN cells.^[^ ^10^, ^11^, ^12^ ^]^ The results show that XEN cells in this study are close to porcine XEN cells derived by Zhang et al,^[^ ^11^ ^]^ which clustered relatively closer to the spherical (Sph, E10‐11) HYPO than late blastocyst (LB, E7‐8) HYPO or day 28 yolk sac (Figure 4M,N). Pluripotency genes (i.e., *SOX2*, *DNMT3B*, and *NANOG*), which are highly expressed in EPI at the early embryo stage,^[^ ^20^ ^]^ are up‐regulated in porcine ESCs (Figure S4A, Supporting Information). While HYPO or XEN‐related genes (i.e., *SOX17*, *GATA6*, and *HNF4A*) that are highly expressed in HYPO,^[^ ^20^ ^]^ are up‐regulated in porcine XEN cells (Figure S4B, Supporting Information). Collectively, these results indicate that we have successfully established and long‐term cultured porcine XEN cells from ESCs by the combination of removing Wnt inhibitors and using the 2FYSC medium (Figure 4O).

### 2.5 Characterizing Chromatin Accessibility Dynamics During the ESC‐to‐XEN Cell Transition

**Figure 5:**
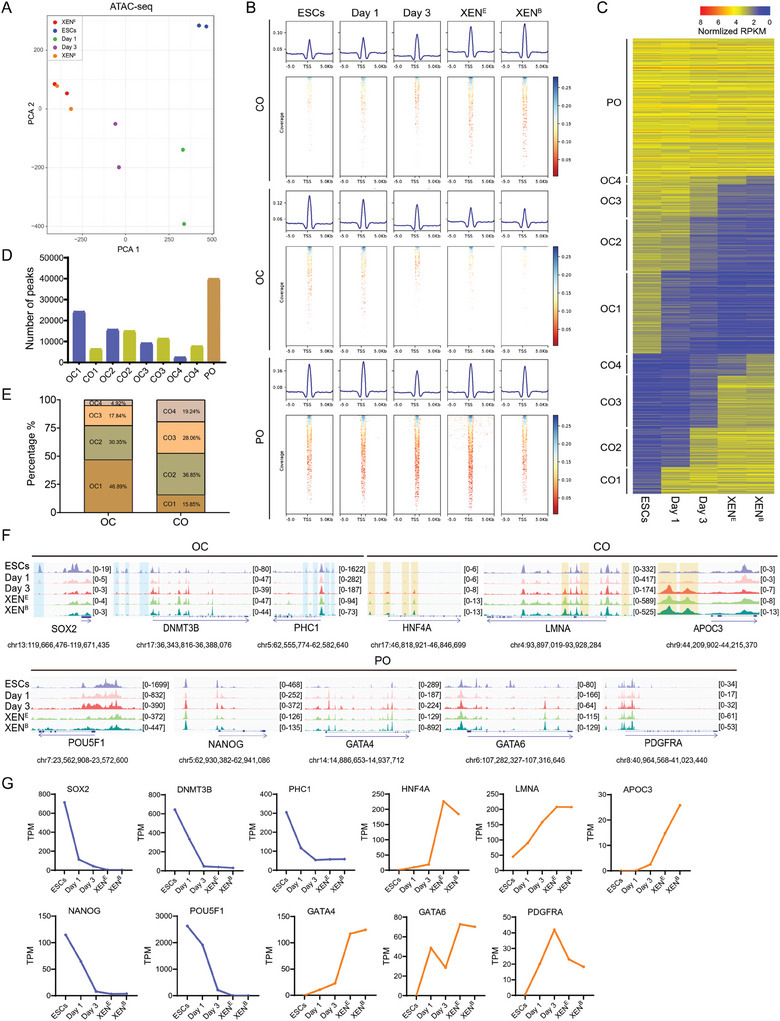
Chromatin accessibility dynamics during porcine ESC‐to‐XEN cell transition. A) PCA analysis by ATAC‐Seq data. B) Density heatmap by ATAC‐Seq. Depicting ATAC‐Seq peaks centered on the peaks within a 5 kb window. C) The global CO/OC and PO status. D) The number of peaks defined as CO, OC, and PO. E) The percentage of CO and OC peaks. F) Representative loci for the OC, CO, and PO peaks at different stages. G) RNA‐Seq analysis of the expression levels (TPM values) of the indicated CO‐ and OC‐related genes.

The chromatin accessibility landscape determines the transcription program of cells and governs the cellular state. To characterize the chromatin accessibility dynamics during ESC‐to‐XEN cell transition, we performed the assay for transposase‐accessible chromatin sequencing (ATAC‐Seq) on porcine ESCs, cells during transition on days 1 and 3, and XEN cells derived from ESCs and blastocysts. PCA analysis shows that the genome accessibility profile undergoes a gradual transition among these cells (**Figure** **5**A). XEN cells transformed from ESCs are consistent with XEN cells from blastocysts in the chromatin patterns (Figure S5A, Supporting Information). We compared the peaks at each locus among these cells at different stages and separated the peaks into three groups: closed in ESCs but open in XEN cells (closed‐to‐open, CO), open in ESCs but closed in XEN cells (open‐to‐closed, OC), permanently open in both ESCs and XEN cells (PO) (Figure 5B,C). To further depict the progression of chromatin accessibility dynamics, we divided the CO and OC peaks into four subgroups (CO1‐4 and OC1‐4), respectively. About 51717 closed and 40833 open peaks were distributed across chromosomes (Figure 5D; Figure S5B, Supporting Information). Counting these peaks showed that OCs outnumber COs during the phase of removing XAV939 and IWR1 (Figure 5D). Compared with ESCs in the 4FIXY medium, cells cultured in the 4FY medium for 1 day closed 46.89% (24250/51717) of loci and opened 15.85% (6473/40833) of loci; cells in the 4FY medium for 3 days further closed 30.35% (15698/51717) of loci and opened 36.85% (15046/40833) of loci (Figure 5D,E), hinting at closing chromatin loci specific to ESCs and opening chromatin loci specific to XEN cells.

We then identified genes specific to the OC or CO group among the peaks opened or closed. Loci for genes that porcine epiblasts highly expressed^[^ ^20^ ^]^ in early‐stage embryos, such as *SOX2*, *DNMT3B*, and *PHC1*, underwent closing, whereas loci for genes highly expressed in porcine HYPO^[^ ^20^ ^]^ in early embryos including *HNF4A*, *APOC3*, and *LMNA* gradually opened (Figure 5F). We further confirm the expression of these genes based on RNA‐Seq data (Figure 5G). Additionally, loci for some common pluripotency genes *POU5F1* and *NANOG*, and HYPO genes *GATA4*, *GATA6*, and *PDGFRA* were opened during the transition (Figure 5F), indicating that Wnt inhibitors including XAV939 and IWR1 regulated their expression without changing the chromatin accessibility. We performed the GO analysis to understand the biological process during the transition. CO peaks are genes that regulate epithelium development, cell differentiation, developmental growth, and endothelial cell migration. OC peaks are near genes involved in stem cell population maintenance, stem cell development, tissue development, and cell fate commitment (Figure S5C,D, Supporting Information). We further performed motif enrichment analysis related to OC and CO peaks. Closed loci during the phase of removing XAV939 and IWR1 for 1 and 3 days (OC1 and OC2) are highly enriched with motifs for TFs from bZIP, FOX, TEA, and ZF families (Figure S5E, Supporting Information). Open loci are mainly enriched with motifs for FOXA2, FOXA1, GATA4, GATA6, GATA1, GATA3, and CTCF (Figure S5E, Supporting Information). Taken together, these data suggest that during the ESC‐to‐XEN cell transition, cells underwent distinct transcription programs apart from common activation of the XEN/HYPO program and suppression of the ESC signature.

### 2.6 Chimeric Contribution of Porcine XEN Cells to Extraembryonic Lineages in Mouse Embryos

**Figure 6:**
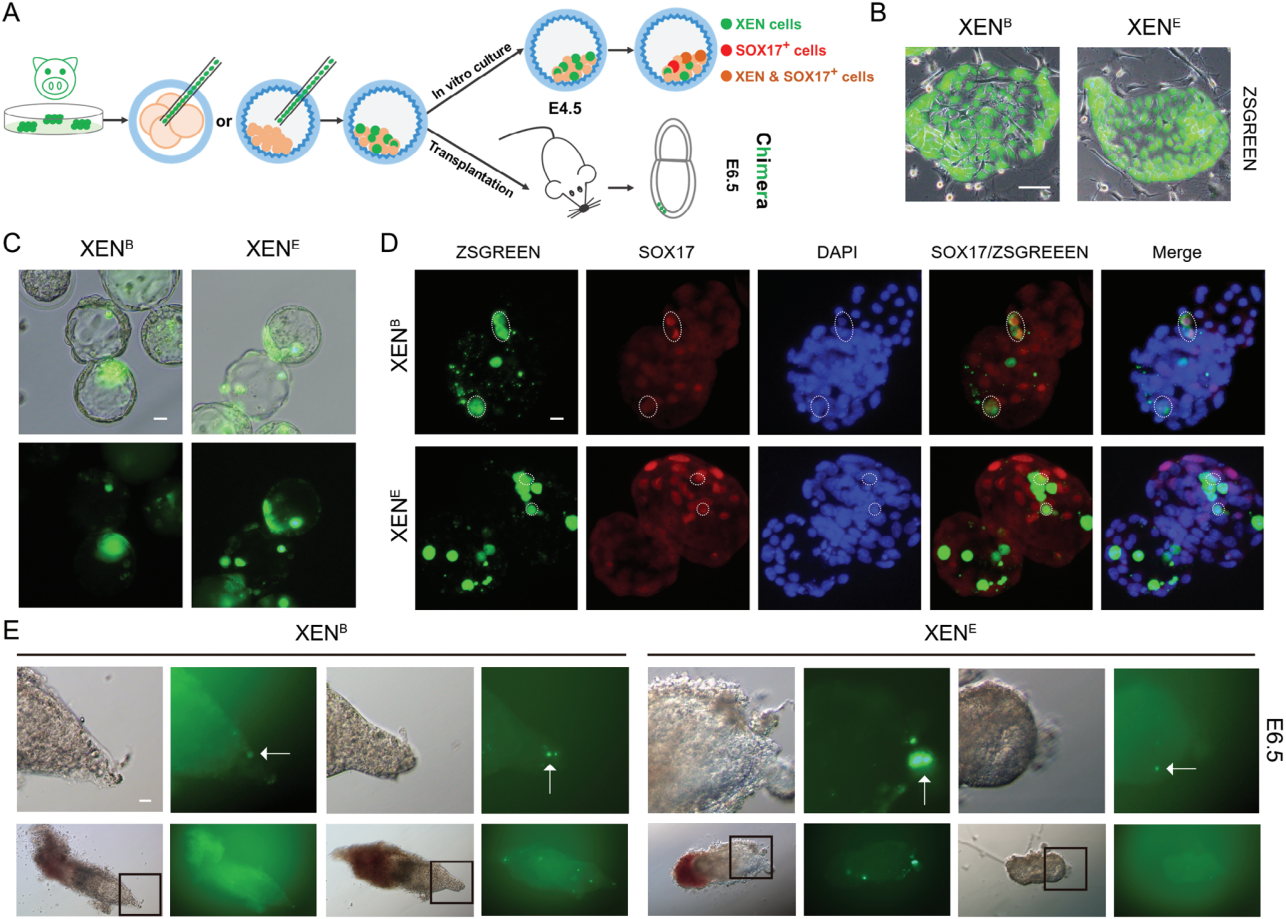
Contribution of porcine XEN cells to chimeras. A) Schematic of chimerism of porcine XEN cells. B) ZSGREEN‐labeled XEN cells from blastocysts (XEN^B^) and ESCs (XEN^E^). Scale bar, 20 µm. C) Representative E4.5 chimeric embryos. Scale bar, 20 µm. D) Representative immunofluorescent staining for SOX17. Scale bar, 20 µm. E) Chimeric contribution of porcine XEN cells into the VE and ParE at E6.5 embryos. Scale bar, 20 µm. XEN^E^: XEN cells from ESCs. XEN^B^: XEN cells from blastocysts.

To evaluate the ability of extraembryonic tissue contributions of porcine XEN cells, we injected ZSGREEN‐labeled porcine XEN cells into mouse 4–8 cell‐stage embryos or blastocysts and in vitro cultured the chimeric embryos until E4.5. Immunofluorescent staining of E4.5 chimeric embryos showed that ZSGREEN‐labeled porcine XEN cells existed in the chimeric embryos and expressed HYPO marker SOX17 (**Figure** **6**A–D), indicating that porcine XEN cells contributed to the HYPO of E4.5 chimeric embryos. We also transplanted chimeric embryos into recipient mice. In E6.5 embryos, porcine XEN cells contributed to the VE and ParE lineages (Figure 6E). These results suggest that porcine XEN cells can contribute to extraembryonic tissues.

## 3 Discussion

Pigs are considered as valuable animal models for regenerative medicine due to their similarities with humans in immunological, physiological, and anatomical traits.^[^ ^1^ ^]^ PSCs represent an attractive source for biomedical research and human therapies. De novo derivation of porcine PSCs from early embryos has been attempted for more than 30 years, but its progress is still far behind PSCs in other species including mice, rats, monkeys, and humans. Recent reports^[^ ^3^, ^4^, ^5^, ^6^, ^7^, ^8^, ^9^ ^]^ have shown the establishment of stable porcine PSCs using various media that contain Wnt inhibitors XAV939 or IWR1. Without XAV939 or IWR1, porcine ESCs^[^ ^8^ ^]^ exhibit differentiation potential; pgEpiSCs^[^ ^6^ ^]^ down‐regulated pluripotency genes and up‐regulated mesoderm or endoderm genes; EPSCs^[^ ^3^, ^4^ ^]^ and EDSCs^[^ ^5^ ^]^ also down‐regulated pluripotency markers. Similarly, in our study, when XAV939 and IWR1 are removed in the ESC culture conditions, pluripotency genes are down‐regulated, suggesting the exit from pluripotency. Our study further elucidates the cell fate determination of porcine ESCs without Wnt inhibitors. Cells without XAV939 and IWR1 become flat and incompact, which is like XEN cells. HYPO‐related genes are up‐regulated in these cells, suggesting that ESCs potentially transform into XEN cells. This will enable the elucidation of mechanisms for porcine pluripotency and embryogenesis.

**Figure 7:**
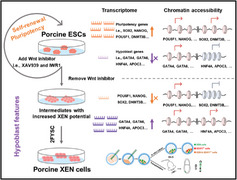
Schematic illustration of the major findings of this study.

Although porcine XEN cells were derived from early embryos using different media, whether XEN cells can be transformed from ESCs without introducing exogenous genes remains unclear. In this work, for the first time, we develop a robust method and an efficient condition, i.e., 2FYSC medium, to support the conversion of XEN cells from porcine ESCs (**Figure** **7**). To develop an efficient culture condition to derive XEN cells from ESCs, we first established the 2FYSC medium to generate and culture XEN cells from porcine early embryos. Cytokines (i.e., EGF and bFGF) and chemical inhibitors (i.e., Y27632, SB431542, and CHIR99021) are applied in this medium. The resulting porcine XEN cells from blastocysts express HYPO markers, i.e., GATA4, GATA6, and SOX17, show similar transcriptomic with in vivo sph HYPO, and contribute to mouse extraembryonic tissues. As shown in Figures 1 and 2, porcine ESCs exit from pluripotency and up‐regulate HYPO/XEN markers without XAV939 and IWR1. To explore whether ESC‐derived cells in 4FY for 3 days can be converted to XEN cells, the 2FYSC medium was used to culture these cells and transform them into XEN cells. More critically, the resulting XEN cells from ESCs express HYPO/XEN markers, exhibit similar transcriptional profiles and chromatin patterns with XEN cells from blastocysts, and contribute to mouse extraembryonic tissues. Establishing porcine XEN cells will provide an in vitro model to explore extraembryonic tissue or HYPO specification and subsequent pre‐ and post‐implantation development. Future studies may focus on the interactions between porcine ESCs and XEN cells and clarify the relationships of epiblast and HYPO in porcine early embryo development.

## 4 Conclusion

In conclusion, the present study shows that Wnt inhibitors, i.e., XAV939 and IWR1, are required for porcine pluripotency maintenance. Without them, porcine ESCs potentially convert toward XEN cells. Additionally, we establish a robust strategy and a culture condition to derive porcine XEN cells from ESCs without genetic interventions. The resulting XEN cells match closely to porcine in vivo sph HYPO and contribute to mouse extraembryonic tissues. This study will facilitate learning of porcine ESC pluripotency and provide a valuable platform to study porcine HYPO specification and the relationship between porcine embryonic and extraembryonic tissues.

## 5 Experimental Section

### Animal Treatment and Ethics Statements

All animal experiments were approved by the Animal Care and Use Committee of Westlake University (AP#22‐035‐4‐PDQ).

### Establishment of Porcine XEN Cells From Blastocysts

The porcine parthenogenetic (PA) blastocysts and ESCs were generated according to the previous report.^[^ ^7^ ^]^ For the derivation of porcine XEN cells, the whole PA blastocysts were digested with 3 mg mL^−1^ pronase (Sigma–Aldrich, P8811) to remove zona pellucida and then cultured in the 2FYSC medium plus 5% FBS on feeder cells until outgrowths appeared. The outgrowths were dissociated with TrypLE Express and reseeded onto mitomycin C‐treated mouse embryonic fibroblast feeder cells in the 2FYSC medium. The cells formed well‐fined colonies ≈3 days later. Cells were cultured at 38.5 °C, 5% O_2_, and 5% CO_2_.

The 2FYSC medium was composed of 1:1 (v/v) mix of Neurobasal medium (Gibco, 21103049) and DMEM/F12 (Gibco, 10565018) supplemented with N2 (Gibco, 17502048) and B27 (Gibco, 17504044) supplements, NEAA (Gibco, 11140050), GlutaMAX (Gibco, 35050061), penicillin/streptomycin (Gibco, 15140122), 5% KOSR, 100 µm 2‐mercaptoethanol (Sigma, M3148), 50 ng mL^−1^ human EGF (Peprotech, AF‐100‐15), 20 ng mL^−1^ human FGF‐basic (Peprotech, 100–18B), 50 µg mL^−1^ 2‐Phospho‐L‐ascorbic acid trisodium salt (Sigma, 49752), 10 µ Y‐27632 (TargetMol, T1725), 1 µm SB431542 (Selleck, S1067), and 2 µ CHIR99021 (Tocris, 4423).

### Culturing Porcine ESCs and XEN Cells

The ESCs and XEN cells were maintained on mitomycin C‐treated mouse embryonic fibroblast feeder cells and enzymatically passaged every 3‐4 days. The ESCs and XEN cells were dissociated as single cells by TrypLE Express (38.5 °C, 5 min), centrifuged (250 g, 5 min), resuspended, and seeded in 4FY and 2FYSC medium, respectively. Cells were passaged at a ratio of 1:3 and cultured at 38.5 °C, 5% O_2_, and 5% CO_2_.

The 4FIXY medium^[^ ^7^ ^]^ was composed of 1:1 (v/v) mix of Neurobasal medium and DMEM/F12 supplemented with N2 and B27 supplements, NEAA, GlutaMAX, penicillin/streptomycin, 5% KOSR, 100 µm 2‐mercaptoethanol, 0.15% FBS (Gibco, 10099141C), 20 ng mL^−1^ human IL‐6 (Peprotech, AF‐200‐06), 20 ng mL^−1^ human sIL‐6 Receptor α (Peprotech, 200–06RC), 20 ng mL^−1^ activin A (Peprotech, 120‐14‐1000), 50 ng mL^−1^ human IGF1 (MCE, HY‐P7018), 2.5 µm XAV939 (Selleck, S1108), 2.5 µm IWR1 (Selleck, S7086), 50 µg mL^−1^ 2‐Phospho‐L‐ascorbic acid trisodium salt and 5 µm Y‐27632.

### Derivation of Porcine XEN Cells From ESCs

Porcine ESCs were digested with Tryple Express into single cells. About 3.3–6.6 × 10^3^ ESCs cm^−2^ were seeded on the mitomycin C‐treated mouse embryonic fibroblast feeder cells and cultured in the 4FIXY medium. On day 1, the medium was replaced with the 4FY medium (4FIXY medium minus XAV939 and IWR1). After the cells reached ≈80% confluence on days 3‐4, they were dissociated with Tryple Express for 5 min at 38.5 °C and passaged to a new feeder cell‐coated 12‐well plate at a 1:2 split ratio. The cells were suspended in the 2FYSC medium. Porcine XEN cells derived from ESCs were generally passaged at a ratio of 1:3 and cultured at 38.5 °C, 5% O_2_, and 5% CO_2_.

### EB Formation

XEN cells were detached using TrypLE Express (38.5 °C, 5 min), plated to ultra‐low attachment multiple well plates in EB formation medium for 7 days, and collected for analysis. Cells were cultured at 38.5 °C, 5% O_2_, and 5% CO_2_. The EB formation medium was composed of 1:1 (v/v) mix of Neurobasal medium and DMEM/F12 supplemented with N2 and B27 supplements, NEAA, GlutaMAX, penicillin/streptomycin, 5% KOSR, 100 µm 2‐mercaptoethanol and 10 µm Y‐27632.

### RNA Extraction and Real‐Time Quantitative PCR

Total RNA was extracted using RNA‐easy Isolation Reagent (Vazyme, R701). Reverse transcription was performed using HiScript II Q RT SuperMix (Vazyme, R222). Real‐time quantitative PCR reactions were performed using ChamQ SYBR Color qPCR Master Mix (Vazyme, Q411) and run on CFX96 Touch Real‐Time PCR Detection System (BIO‐RAD). Relative expression values were normalized to EF1α. Primers in this study are shown in Table S1 (Supporting Information).

### Immunofluorescent staining

Cells or embryos were fixed with 4% paraformaldehyde for ≈45 min at room temperature, washed in PBS, and permeabilized with 0.2% Triton X‐100 in PBS for 20 min. Samples were then blocked with the blocking buffer at room temperature for ≈45 min. Cells were incubated in primary antibodies overnight at 4 °C, washed three times for 15 min with PBS, and incubated with fluorescent‐dye conjugated secondary antibodies diluted in secondary antibody dilution buffer for 1 h at room temperature. Finally, samples were washed three times with PBS and counterstained with DAPI at room temperature for ≈3 min. Primary antibodies were as follows: anti‐GATA6 (5851, Cell Signaling Technology), anti‐SOX17 (AF1924, R&D), anti‐GATA4 (sc‐25310, Santa Cruz Biotechnology), anti‐SOX2 (MAB2018, R&D), anti‐GATA3 (5852, Cell Signaling Technology), anti‐CDX2 (ab76541, Abcam). Secondary antibodies include Alexa 488 goat anti‐rabbit (4412, Cell Signaling Technology), Alexa 594 donkey anti‐goat (ab150132, Abcam), Alexa 488 goat anti‐mouse (4408, Cell Signaling Technology), and Alexa 594 goat anti‐rabbit (8889, Cell Signaling Technology). Embryos were imaged in a glass slide under a coverslip.

### Flow Cytometry

Porcine ESCs were genetically engineered with SOX2‐promoter‐GFP (GFP) to generate the fluorescence reporter cell lines. These ESCs in the 4FIXY medium and cells in the 4FY medium for 3 days were digested into single cells by Tryple Express, suspended in DPBS, and analyzed by flow cytometry (Beckman Coulter, CytoFLEX SRT).

### Karyotype Analysis

Before karyotype analysis, cells were treated with 4 µg mL^−1^ colchicine in the culture medium for ≈2 h. The cells were digested, centrifuged, resuspended with 0.075 m KCl hypotonic solution, and incubated at 37 °C for 30 min. After that, cells were fixed with a precooled 3:1 (v/v) mix of methanol and acetic acid, and this step was repeated three times. The resuspended cells were dropped on precooled slides, dried, and stained with Giemsa.

### Doubling Time Analysis

The doubling time of the cells was calculated using the online calculator (https://www.calculatorultra.com/en/tool/cell‐doubling‐time‐calculator.html).

### Bulk RNA‐Seq and Analysis

About 1 µg RNA was used for constructing the library and sequenced on an Illumina Novaseq PE150 platform. Raw data was processed using Trim Galore; Clean reads were aligned to the Sus scrofa 11.1 reference genome with HISAT2^[^ ^22^ ^]^ and the expression was calculated by featureCount^[^ ^23^ ^]^ with Ensembl annotations (v112). To make a comparable analysis with previously published data, batch effects and other unwanted variations were removed in the expression data from GSE112380,^[^ ^20^ ^]^ GSE140414,^[^ ^10^ ^]^ GSE183270,^[^ ^11^ ^]^ and GSE128149^[^ ^12^ ^]^ using sva.^[^ ^24^ ^]^ DEGs were performed with DESeq2.^[^ ^25^ ^]^ The GO and KEGG enrichment analysis was performed using clusterProfiler.^[^ ^26^ ^]^ The plot was generated using R software (v.4.2.2) package“pheatmap”(v.1.0.12)^[^ ^27^ ^]^ through Hiplot Pro (https://hiplot.com.cn/), a comprehensive web service for biomedical data analysis and visualization.

### ATAC‐Seq and Analysis

About 10^5^ cells were used for constructing the library using the Hyperactive ATAC‐Seq Library Prep Kit for Illumina (Vazyme Biotech Co. Ltd, TD711). The initial quality of raw data was assessed using FastQC to evaluate sequencing quality and adapter contamination. The raw reads were trimmed for sequencing adapters using Trim Galore and the clean reads were aligned to the Sus scrofa 11.1 reference using bwa.^[^ ^28^ ^]^ Post‐alignment, the resulting BAM files were sorted and deduplicated using samtools and picard. Normalized signal tracks were generated using deeptools (v3.5.5)^[^ ^29^ ^]^ for downstream visualization and analysis. Normalized signals for POU5F1, SOX2, NANOG, DNMT3B, PHC1, LMNA, APOC3, GATA4, GATA6, and PDGFRA and Log (normalized signal, e) for HNF4A were shown as indicated. MACS3 (v3.0.2)^[^ ^30^ ^]^ was used to identify accessible chromatin regions by calling peaks on the ATAC‐Seq data. To analyze the differencially accessible regions (DARs) between samples, consensus peaks were merged with bedtools,^[^ ^31^ ^]^ and the count of peaks was calculated by featureCount.^[^ ^23^ ^]^ Downstream DARs were performed with DESeq2.^[^ ^25^ ^]^ HOMER^[^ ^32^ ^]^ was used to annotate the peaks and perform the motif analysis. The GO enrichment analysis of genes located in DARs was performed using clusterProfiler.^[^ ^26^ ^]^ The genomic view of DARs was generated by IGV.^[^ ^33^ ^]^

### Microinjection of Porcine XEN Cells Into Mouse Embryos

Mouse embryo collection and culture were conducted as described.^[^ ^34^, ^35^ ^]^ Ten to fifteen ZSGREEN‐labeled porcine XEN cells were injected into 4–8 cell‐stage embryos or blastocysts. The chimeric embryos were in vitro cultured and immunostained to detect the SOX17 expression at E4.5. The in vitro culture medium was a 1:1 mixture of 2FYSC and KSOM. Additionally, the chimeric embryos were cultured and transferred into the uterus of pseudopregnant mice to detect the developmental fate of porcine XEN cells in E6.5 chimeric embryos. ≈20 chimeric embryos per recipient were transferred into the uterus of pseudopregnant females.

### Statistical Analysis

The data were presented as the mean ± SD. The numbers of sample sizes for each experiment were indicated in the figure legend. Unpaired two‐tailed Student's *t* test was used to assess statistically significant in real‐time quantitative PCR. One‐way ANOVA was used to assess statistically significant in doubling time analysis. *p* value < 0.05 is considered significant. ^*^ *p*<0.05; ^**^ *p*<0.01; ^***^ *p*<0.001; ^****^ *p*<0.0001. GraphPad Prism 10 (GraphPad Software, Inc., USA) was applied for statistical analysis.

## Conflict of Interest

The authors declare no conflict of interest.

## Author Contributions

H.W. and L.Z. contributed equally to this work. D.P., J.X., and H.W. designed and supervised the study. H.W. and J.X. performed the experiments and analyzed the data. J.X. and Z.W. performed porcine oocyte collection and maturation experiments. L.Z. performed the bioinformatic analysis. H.W. and J.X. wrote the original draft, and D.P. revised the manuscript. All authors read and approved the final manuscript.

## Supporting information



Supporting Information

## Data Availability

The data that support the findings of this study are available from the corresponding author upon reasonable request.
