# Supplementary material for: Wnt Inhibition Safeguards Porcine Embryonic Stem Cells From the Acquisition of Extraembryonic Endoderm Cell Fates
Source: Adv Sci (Weinh). 2025 Mar 10;12(17):2416802. doi: 10.1002/advs.202416802 (PMC12061302; doi:10.1002/advs.202416802)
Supplement: Supplementary file 1 — Supporting Information [file ADVS-12-2416802-s001.docx]

**Supplementary Information**

**Wnt inhibition safeguards porcine embryonic stem cells from the acquisition of extraembryonic endoderm cell fates**

*Hanning Wang, Liang Zhong, Zhuangfei Wang, Jinzhu Xiang*, Duanqing Pei**

H. Wang, Z. Wang, J. Xiang, D. Pei

Laboratory of Cell Fate Control, School of Life Sciences

Westlake University

Hangzhou, 310030, China

E-mail: xiangjinzhu@westlake.edu.cn; peiduanqing@westlake.edu.cn

L. Zhong

Hebei Provincial Key Laboratory of Basic Medicine for Diabetes

The Shijiazhuang Second Hospital

Shijiazhuang, 050051, China

D. Pei

Westlake Laboratory of Life Sciences and Biomedicine

Hangzhou, 310030, China

**Table S1. Primers for real-time quantitative PCR analyses used in this study.**

| Gene | Forward (5'-3') | Reverse (5'-3') |  |
| --- | --- | --- | --- |
| EF1α | AATGCGGTGGGATCGACAAA | CACGCTCACGTTCAGCCTTT |  |
| POU5F1 | TGAGGCTTTGCAGCTCAGTT | TCTCCAGGTTGCCTCTCACT |  |
| SOX2 | CATCAACGGTACACTGCCTCTC | ACTCTCCTCCCATTTCCCTCTTT |  |
| NANOG | CCTACAATCCAGCTCTTTGG | CTCAGGCATTGGTGAAGATT |  |
| OTX2 | CGGAGTCCAGGGTTCAGGTA | ACTGGCCACTTGTTCCACTC |  |
| SOX17 | CGCACGGAGTTTGAACAATA | CAGACGTCGGGGTAGTTACAG |  |
| GATA4 | CGACACCCTAATCTCGATATGTTT | GTGGTGGTCTGGCAGTTGG |  |
| GATA6 | ATCACCATCACCACCCAAGT | CGCGACTCTGTAGACTGTGC |  |
| COL4A1 | GTGCATGCGGAGAACATGAC | AGGGTGTGTTAGTTACGCGG |  |
| PDGFRA | CAGGTTGGAGGGAGATGGAC | AGTTGCGGAGGTTGGATT |  |
| FOXA2 | ATAAGGAGGGCAAGGGAAAA | AGTCAAAATTCGCAGGTGCT |  |
| APOE | GAAGATGAGGGTTCTGTGGGTT | TGGGTGACCTTGGTGCTGA |  |
| CLDN6 | CCTTCATCGGCAACAGCATC | GCCAGCAGCGAGTCATACACC |  |
| AFP | CACCTTTCCAGGTTCCAGAA | AAGGGGTGCCTTCTTGCTAT |  |
| SNAIL | TTTTCAGCAGCCCTATGACC | CCAGGAGAGAGTCCCAGATG |  |
| PLAU | AAGGGCTCTGACATTCCATG | CCGGCTCTTACACTGACACA |  |
| SPARC | | GGACCATCAGTCCTCTGGAA | AGTTCTGCGTCTCCCAAAGA |


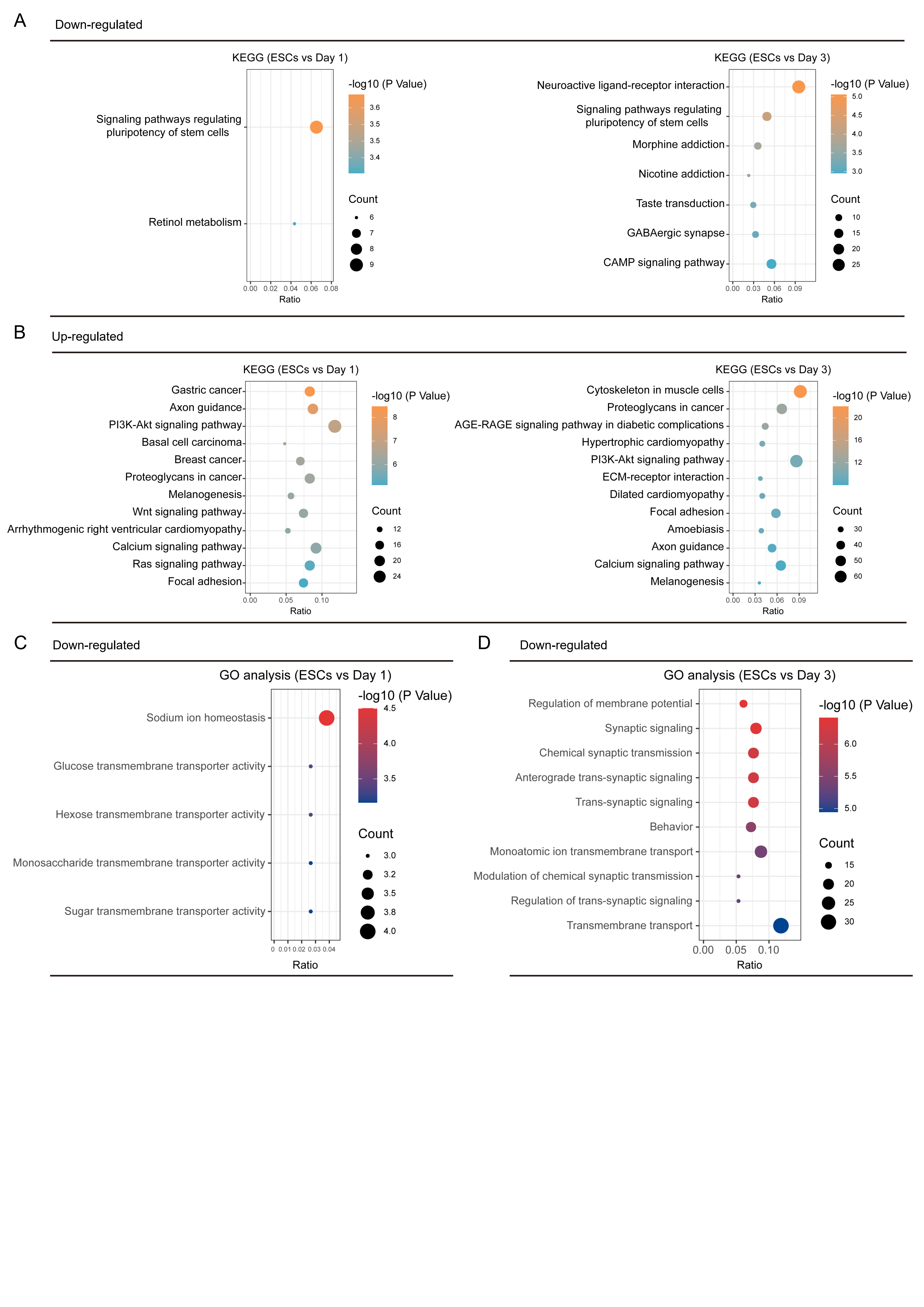


**Figure S1. GO and KEGG enrichment analysis between ESCs and cells in the 4FY medium.** A) KEGG enrichment of downregulated genes in 4FY for 1 and 3 days. B) Top KEGG enrichment of upregulated genes in 4FY for 1 and 3 days. C) GO analysis of downregulated genes in 4FY for 1 day. D) GO analysis of downregulated genes in 4FY for 3 days.


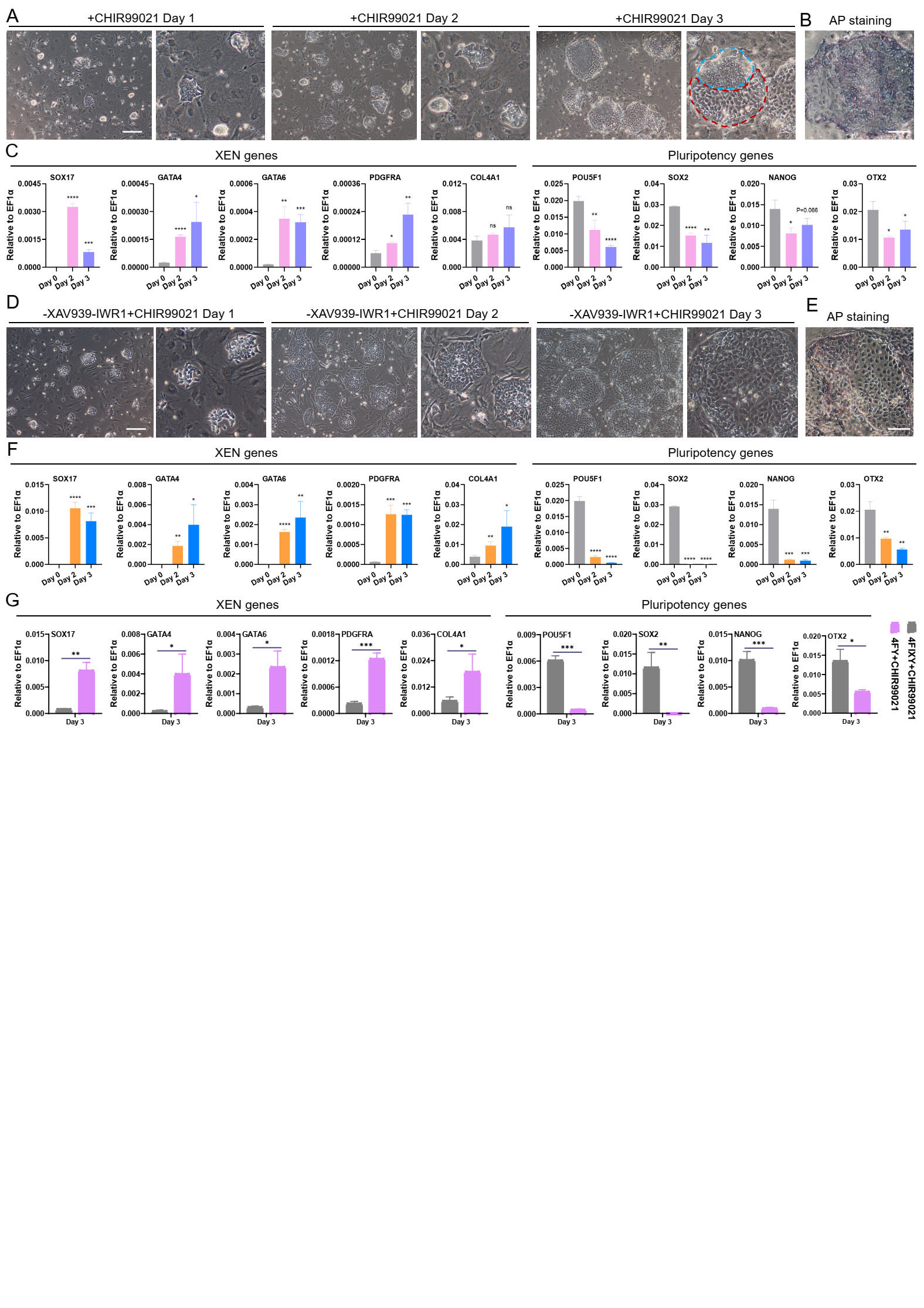


**Figure S2.** **CHIR99021 promotes the conversion of XEN cells from ESCs.** A) Representative morphology of cells cultured in the 4FIXY plus CHIR99021 culture condition. Scale bar, 200 μm. B) AP staining in 4FIXY plus CHIR99021 medium for 3 days. Scale bar, 100 μm. C) Real-time quantitative PCR data of XEN and pluripotency markers following 0 (ESCs), 2, and 3 days of exposure to the 4FIXY plus CHI99021 culture condition. n=3. The data are presented as the mean ± SD. Day 2 vs. Day 0; Day 3 vs. Day 0. * p<0.05; ** p<0.01; *** p<0.001; **** p<0.0001. The p values were calculated using Student’s t. D) Representative morphology of cells cultured in the 4FY plus CHIR99021 culture condition. Scale bar, 200 μm. E) AP staining of cells in 4FY plus CHIR99021 medium for 3 days. Scale bar, 100 μm. F) Real-time quantitative PCR data of XEN and pluripotent markers following 0 (ESCs), 2, and 3 days of exposure to the 4FY plus CHIR99021 culture condition. n=3. The data are presented as the mean ± SD. Day 2 vs. Day 0; Day 3 vs. Day 0. * p<0.05; ** p<0.01; *** p<0.001; **** p<0.0001. The p values were calculated using Student’s t. G) Real-time quantitative PCR data of XEN and pluripotent markers following 3 days of exposure to the 4FY plus CHIR99021 and 4FIXY plus CHIR99021 culture conditions. n=3. The data are presented as the mean ± SD. * p<0.05; ** p<0.01; *** p<0.001. The p values were calculated using Student’s t.


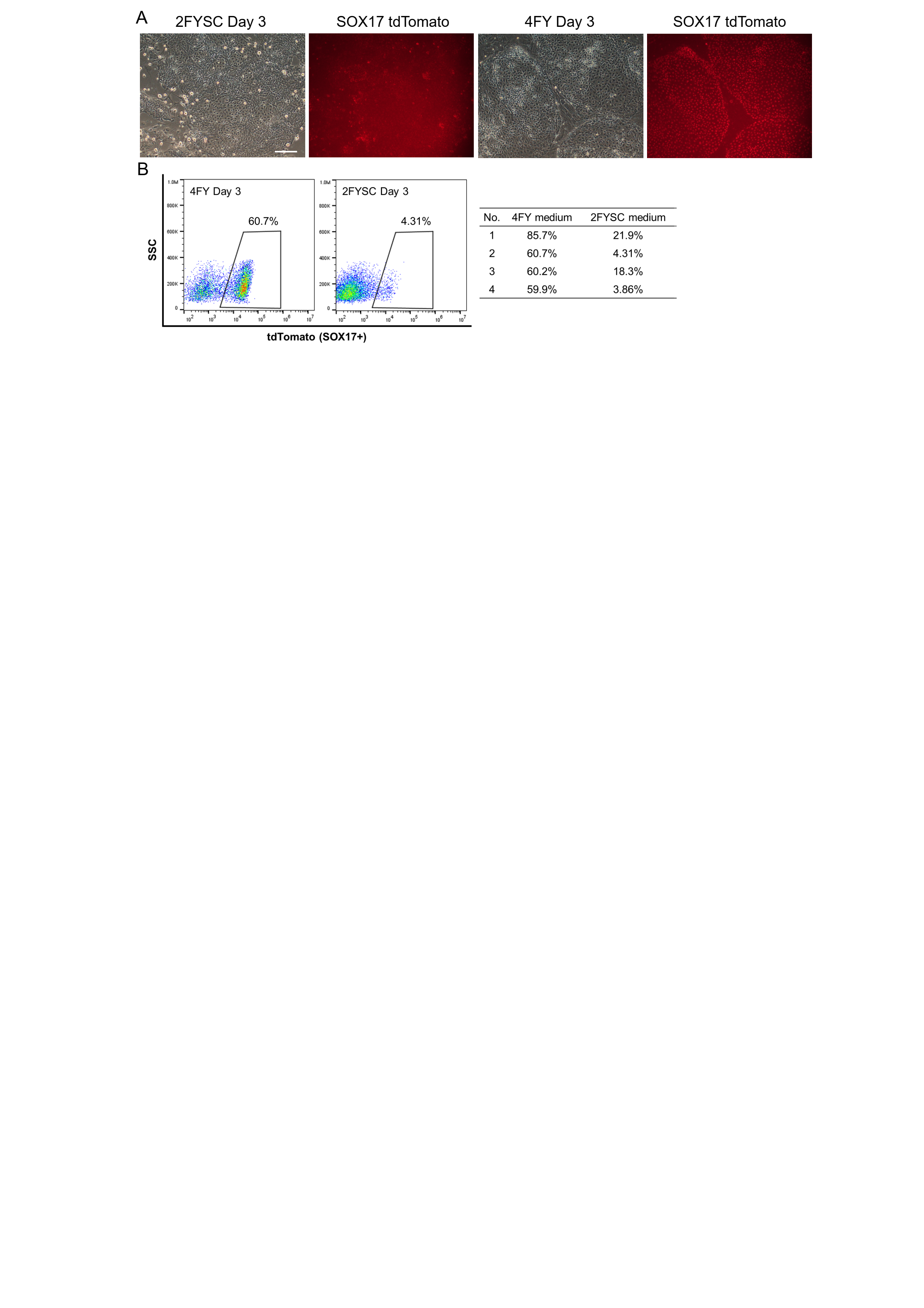


**Figure S3.** **Culturing porcine ESCs in 2FYSC and 4FY medium.** A) Representative morphology of porcine ESCs cultured in 2FYSC and 4FY medium. Scale bar, 200 μm. B) Flow cytometric analysis of SOX17 positive cells following 3 days of treatment in 2FYSC and 4FY medium.


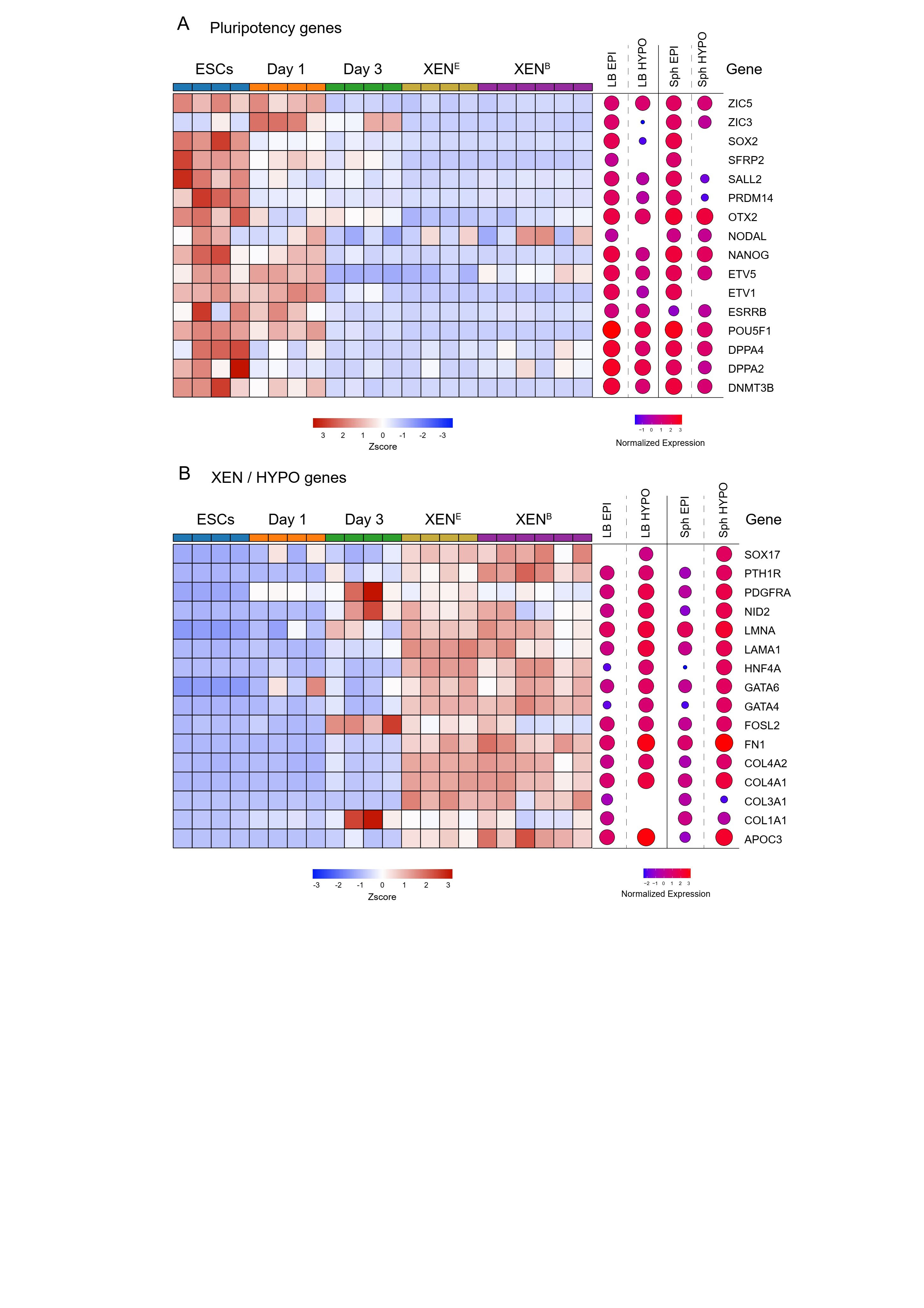


**Figure S4.** **The gene expression in various cell types and porcine early embryos.** A) Heatmap and bubble chart showing pluripotency gene expression in porcine ESCs, cells in the 4FY medium, and porcine early embryos^[1]^. B) Heatmap and bubble chart displaying XEN or HYPO gene expression in porcine ESCs, cells in the 4FY medium, and porcine early embryos^[1]^. XEN^E^: XEN cells from ESCs. XEN^B^: XEN cells from blastocysts. LB: late blastocyst; Sph: spherical embryo; EPI: epiblast; HYPO: hypoblast.


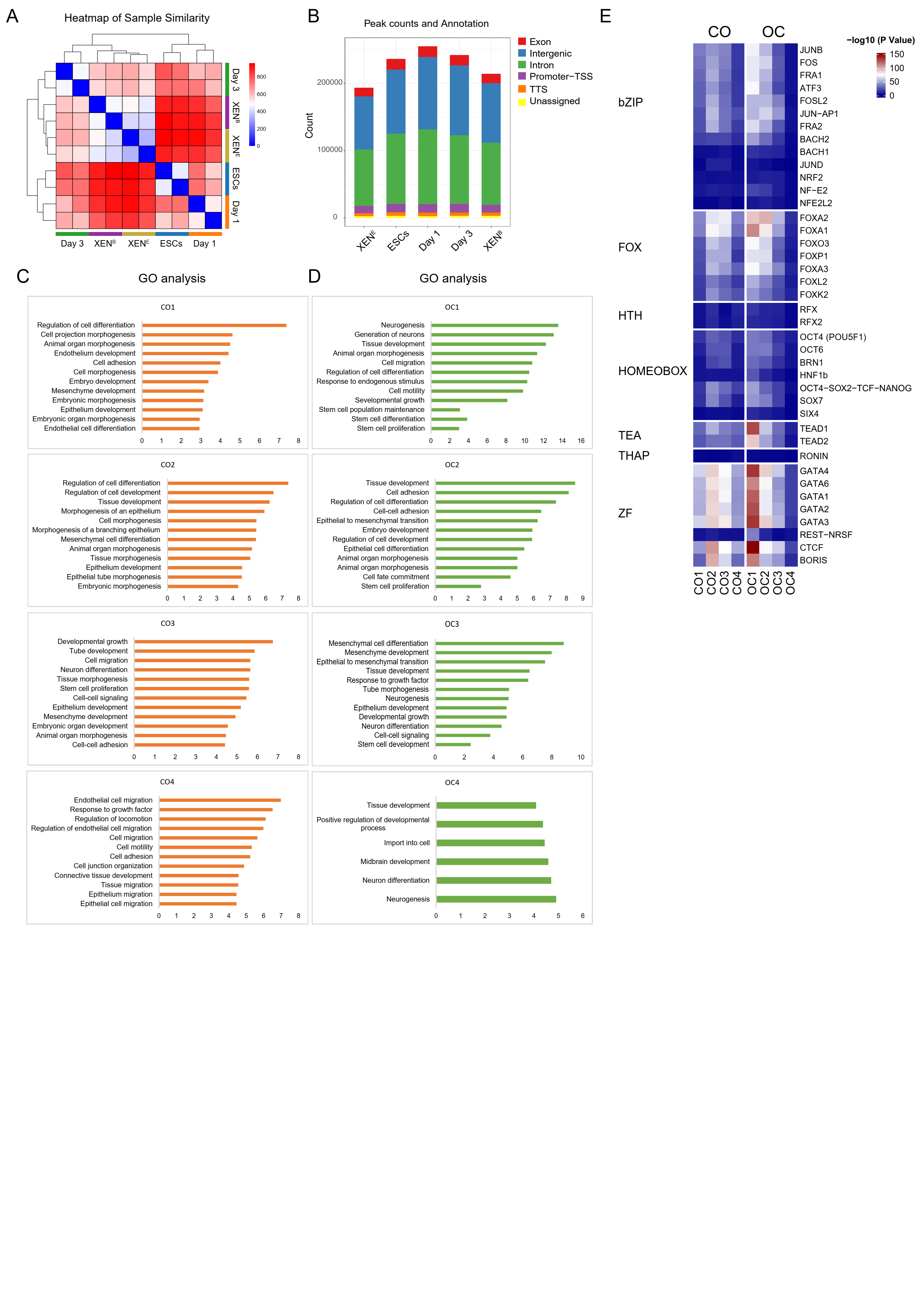


**Figure S5. The analysis for ATAC-Seq.** A) Heatmap showing the sample similarities. B) Distribution of functional regions. C, D) GO enrichment of genes related to CO and OC peaks. E) Heatmap showing the motif enrichment of the CO and OC peak. TF families are indicated on the left.

**Reference**

[1] P. Ramos-Ibeas, F. Sang, Q. Zhu, W. W. C. Tang, S. Withey, D. Klisch, L. Wood, M. Loose, M. A. Surani, R. Alberio, *Nat Commun*. **2019**, *10*, 500.
